# Case of Exomphalos, Successfully Treated by Ligature

**Published:** 1826-11

**Authors:** G. J. Hunter


*Case of Exomphalos, successfully treated by Ligature.*
By Mr. G. J. Hunter.

The following case is not uncommon, neither is the treatment original; but, as it adds another to the successful instances already recorded, you may perhaps think fit to insert it in your Journal.

The male child of William Gibbs, aged two months, was brought to me on the 8th of November, 1825, in consequence of an exomphalos, which had appeared about a month, and was then as large as a nutmeg. I carefully applied pressure in the usual manner, which I had always before found successful; but, notwithstanding all my efforts, the hernia gradually increased in size, and, on the 12th of January following, it was larger than a walnut.

The mother, then getting impatient, carried the child to Dr. Scudamore, who recommended me to use a strong decoction of oak-bark, as proposed by Mr. Lizars. This I accordingly directed to be constantly applied, with the addition of two ounces of alum to a quart, but the tumor still enlarged; and, therefore, on the 31st of January, I carefully returned the intestine, (which Dr. Scudamore retained within the cavity of the abdomen with his finger,) and passed a ligature, composed of four pieces of waxed silk, round the integuments, and as near as possible to the abdominal opening. The part included in the ligature rapidly sphacelated, and on the 14th of February it came away, leaving a healthy granulating surface, which cicatrised by the 12th March, at which time the cure was complete.

There was very little constitutional irritation, and the only medicine administered was a gentle aperient twice during the progress of the cure.

I shall only observe further, that the intestine never protruded after the ligature was applied, and the child (whom I saw yesterday) continues well.

*Margate; October 8th,* 1826.

